# *catena*-Poly[[[triaqua­copper(II)]-μ-pyridine-2,3-dicarboxyl­ato-κ^3^ *N*,*O* ^2^:*O* ^3^] monohydrate]

**DOI:** 10.1107/S1600536808025439

**Published:** 2008-09-06

**Authors:** Lujiang Hao, Chunhua Mu, Binbin Kong

**Affiliations:** a College of Food and Biological Engineering, Shandong Institute of Light Industry, Jinan 250353, People’s Republic of China; b Maize Research Insitute, Shandong Academy of Agricultural Science, Jinan 250100, People’s Republic of China

## Abstract

In the title compound, {[Cu(C_7_H_3_NO_4_)(H_2_O)_3_]·H_2_O}_*n*_, the Cu^II^ ion is bonded to three water mol­ecules, one *N*,*O*-bidentate pyridine-2,3-dicarboxyl­ate dianion and one O-bonded symmetry-generated dianion, resulting in a distorted CuNO_5_ octa­hedral geometry. The bridging ligand results in an infinite chain. A network of O—H⋯O hydrogen bonds helps to establish the crystal structure.

## Related literature

For background, see: Serre *et al.* (2005 ▶).
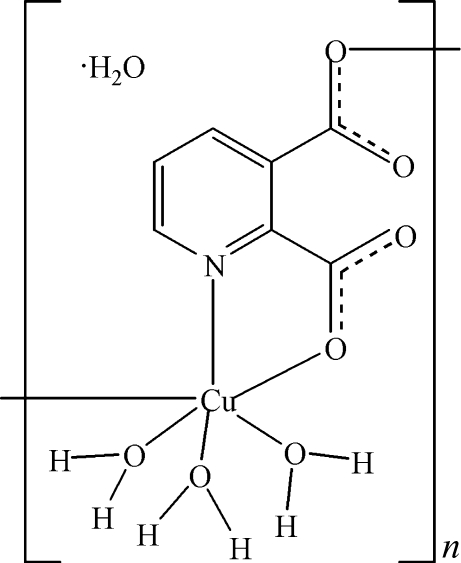


## Experimental

### 

#### Crystal data

[Cu(C_7_H_3_NO_4_)(H_2_O)_3_]·H_2_O
*M* *_r_* = 300.71
Monoclinic, 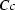

*a* = 8.513 (3) Å
*b* = 17.983 (3) Å
*c* = 7.493 (3) Å
β = 114.486 (10)°
*V* = 1043.9 (6) Å^3^
*Z* = 4
Mo *K*α radiation
μ = 2.13 mm^−1^
*T* = 296 (2) K
0.40 × 0.28 × 0.22 mm

#### Data collection

Bruker APEXII CCD diffractometer
Absorption correction: multi-scan (*SADABS*; Bruker, 2004 ▶) *T*_min_ = 0.484, *T*_max_ = 0.652
2686 measured reflections
1322 independent reflections
1310 reflections with *I* > 2σ(*I*)
*R*_int_ = 0.028

#### Refinement

*R*[*F* ^2^ > 2σ(*F* ^2^)] = 0.030
*wR*(*F* ^2^) = 0.089
*S* = 1.00
1322 reflections
180 parameters
14 restraints
H atoms treated by a mixture of independent and constrained refinement
Δρ_max_ = 0.61 e Å^−3^
Δρ_min_ = −0.60 e Å^−3^
Absolute structure: Flack (1983 ▶), 290 Friedel pairs
Flack parameter: 0.05 (3)

### 

Data collection: *APEX2* (Bruker, 2004 ▶); cell refinement: *SAINT-Plus* (Bruker, 2004 ▶); data reduction: *SAINT-Plus*; program(s) used to solve structure: *SHELXS97* (Sheldrick, 2008 ▶); program(s) used to refine structure: *SHELXL97* (Sheldrick, 2008 ▶); molecular graphics: *SHELXTL* (Sheldrick, 2008 ▶); software used to prepare material for publication: *SHELXTL*.

## Supplementary Material

Crystal structure: contains datablocks global, I. DOI: 10.1107/S1600536808025439/hb2747sup1.cif

Structure factors: contains datablocks I. DOI: 10.1107/S1600536808025439/hb2747Isup2.hkl

Additional supplementary materials:  crystallographic information; 3D view; checkCIF report

## Figures and Tables

**Table 1:** Selected bond lengths (Å)

Cu1—O7	2.061 (3)
Cu1—O6	2.068 (3)
Cu1—O3	2.071 (3)
Cu1—O1	2.119 (4)
Cu1—O5	2.178 (5)
Cu1—N1	2.187 (4)

**Table 2:** Hydrogen-bond geometry (Å, °)

*D*—H⋯*A*	*D*—H	H⋯*A*	*D*⋯*A*	*D*—H⋯*A*
O5—H3*W*⋯O4^i^	0.82 (9)	1.93 (8)	2.735 (6)	167 (8)
O5—H4*W*⋯O6^ii^	0.82 (2)	2.35 (8)	2.966 (5)	132 (10)
O6—H5*W*⋯O5^iii^	0.82 (7)	2.29 (7)	2.966 (5)	140 (9)
O7—H7*W*⋯O2^iv^	0.83 (8)	1.90 (9)	2.720 (5)	169 (8)
O7—H8*W*⋯O2^ii^	0.83 (8)	2.03 (4)	2.825 (5)	162 (11)

Symmetry codes: (i) 
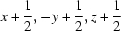
; (ii) 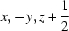; (iii) 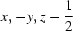; (iv) 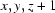.

**Fig. 1.**
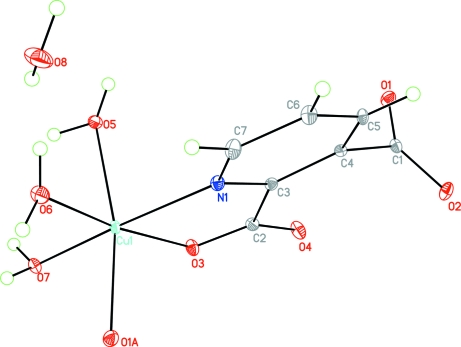
The molecular structure of (I), drawn with 30% probability displacement ellipsoids for the non-hydrogen atoms. Atoms labeled with i are at the symmetry position (x - 1/2,-y + 1/2,z - 1/2).

**Fig. 2.**
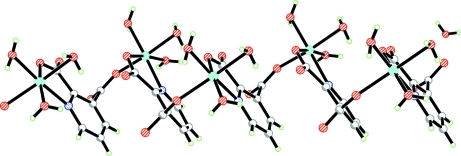
Part of a polymeric chain in (I).

## supplementary crystallographic information

### Comment

In recent years, carboxylic acids have been widely used as polydentate ligands, which can coordinate to transition or rare earth ions yielding complexes with interesting properties in biological systems (e.g. Serre *et al.*, 2005). Herein, we report the synthesis and structure of the title compound, (I).

As shown in Fig. 1, the Cu^II^ ion in (I) is hexacoordinated with five oxygen atoms and one nitrogen atom, exhibiting a slightly distorted octahedral geometry (Table 1). The pyridine-2,3-dicarboxyalto ligand affords the pyridine N and one carboxylato oxygen atom in chelating coordination mode and a symmetry-generated ligand bonds from its carboxylate O-atom. The bridging ligand links neighboring copper(II) ions into a chain (Fig. 2). Extensive hydrogen bonding (Table 2) *via* hydrogen bonds between carboxylate oxygen atoms of pyridine-2,3-dicarboxyalte and the uncoordinated water molecules or coordinated aqua ligands, giving rise to a three-dimensional network.

### Experimental

A mixture of copper(II) chloride (0.5 mmol), pyridine-2,3-dicarboxylic acid (1 mmol), sodium hydroxide (1 mmol), H_2_O (8 ml), and ethanol (8 ml) in a 25 ml Teflon-lined stainless steel autoclave was kept at 423 K for three days. Blue blocks of (I) were obtained after cooling to room temperature with a yield of 16%. Anal calc. for C_7_H_11_CuNO_8_: C 27.93, H 2.99, N 4.66%; found: C 27.89, H 2.92, N 4.63%.

### Refinement

The O-bound H atoms were located in a difference map and their positions were freely refined with a fixed U_iso_ value. This has led to some extremely short intermolecular H···H contacts and the location of the water H atoms should be regarded as less certain. All the C-bound H atoms were placed in calculated positions with C—H = 0.93Å and refined as riding with *U*_iso_(H) = 1.2*U*_eq_(C).

### Crystal data

**Table:**

[Cu(C_7_H_3_NO_4_)(H_2_O)_3_]·H_2_O	*F*(000) = 612
*M**_r_* = 300.71	*D*_x_ = 1.913 Mg m^−^^3^
Monoclinic, *C**c*	Mo *K*α radiation, λ = 0.71073 Å
Hall symbol: C -2yc	Cell parameters from 2692 reflections
*a* = 8.513 (3) Å	θ = 2.9–28.1°
*b* = 17.983 (3) Å	µ = 2.13 mm^−^^1^
*c* = 7.493 (3) Å	*T* = 296 K
β = 114.486 (10)°	Block, blue
*V* = 1043.9 (6) Å^3^	0.40 × 0.28 × 0.22 mm
*Z* = 4	

### Data collection

**Table:**

Bruker APEXII CCD diffractometer	1322 independent reflections
Radiation source: fine-focus sealed tube	1310 reflections with *I* > 2σ(*I*)
graphite	*R*_int_ = 0.028
ω scans	θ_max_ = 26.0°, θ_min_ = 2.9°
Absorption correction: multi-scan (SADABS; Bruker, 2004)	*h* = −10→10
*T*_min_ = 0.484, *T*_max_ = 0.652	*k* = −22→22
2686 measured reflections	*l* = −4→9

### Refinement

**Table:**

Refinement on *F*^2^	Hydrogen site location: difmap and geom
Least-squares matrix: full	H atoms treated by a mixture of independent and constrained refinement
*R*[*F*^2^ > 2σ(*F*^2^)] = 0.030	*w* = 1/[σ^2^(*F*_o_^2^) + (0.079*P*)^2^ + 0.0702*P*] where *P* = (*F*_o_^2^ + 2*F*_c_^2^)/3
*wR*(*F*^2^) = 0.089	(Δ/σ)_max_ < 0.001
*S* = 1.00	Δρ_max_ = 0.61 e Å^−^^3^
1322 reflections	Δρ_min_ = −0.60 e Å^−^^3^
180 parameters	Extinction correction: SHELXL97 (Sheldrick, 2008), Fc^*^=kFc[1+0.001xFc^2^λ^3^/sin(2θ)]^-1/4^
14 restraints	Extinction coefficient: 0.018 (2)
Primary atom site location: structure-invariant direct methods	Absolute structure: Flack (1983), 290 Friedel pairs
Secondary atom site location: difference Fourier map	Flack parameter: 0.05 (3)

### Special details

**Table:**

Geometry. All e.s.d.'s (except the e.s.d. in the dihedral angle between two l.s. planes) are estimated using the full covariance matrix. The cell e.s.d.'s are taken into account individually in the estimation of e.s.d.'s in distances, angles and torsion angles; correlations between e.s.d.'s in cell parameters are only used when they are defined by crystal symmetry. An approximate (isotropic) treatment of cell e.s.d.'s is used for estimating e.s.d.'s involving l.s. planes.
Refinement. Refinement of *F*^2^ against ALL reflections. The weighted *R*-factor *wR* and goodness of fit *S* are based on *F*^2^, conventional *R*-factors *R* are based on *F*, with *F* set to zero for negative *F*^2^. The threshold expression of *F*^2^ > σ(*F*^2^) is used only for calculating *R*-factors(gt) *etc*. and is not relevant to the choice of reflections for refinement. *R*-factors based on *F*^2^ are statistically about twice as large as those based on *F*, and *R*- factors based on ALL data will be even larger.

### Fractional atomic coordinates and isotropic or equivalent isotropic displacement parameters (Å^2^)

**Table:**

	*x*	*y*	*z*	*U*_iso_*/*U*_eq_	
Cu1	0.00453 (5)	0.118674 (19)	0.25189 (5)	0.0226 (2)	
C1	−0.1893 (6)	0.1125 (2)	−0.2173 (8)	0.0257 (10)	
C2	0.0162 (7)	0.2776 (2)	0.2417 (8)	0.0261 (9)	
C3	0.1909 (5)	0.2542 (2)	0.2398 (6)	0.0220 (8)	
C4	0.3188 (5)	0.3044 (2)	0.2468 (6)	0.0224 (8)	
C5	0.4612 (5)	0.2816 (2)	0.2256 (6)	0.0222 (9)	
H5	0.5443	0.3154	0.2266	0.027*	
C6	0.4781 (6)	0.2094 (3)	0.2034 (7)	0.0325 (10)	
H6	0.5747	0.1922	0.1878	0.039*	
C7	0.3554 (7)	0.1592 (3)	0.2028 (8)	0.0350 (10)	
H7	0.3719	0.1088	0.1890	0.042*	
N1	0.2118 (5)	0.18178 (19)	0.2218 (6)	0.0262 (7)	
O1	−0.1400 (5)	0.0911 (2)	−0.0470 (5)	0.0356 (8)	
O2	−0.2392 (5)	0.07157 (19)	−0.3667 (5)	0.0363 (7)	
O3	−0.0834 (4)	0.22671 (17)	0.2399 (6)	0.0323 (7)	
O4	−0.0130 (6)	0.34452 (16)	0.2387 (9)	0.0401 (8)	
O5	0.1420 (5)	0.12328 (17)	0.5699 (7)	0.0311 (9)	
O6	0.1266 (5)	0.01799 (18)	0.2664 (7)	0.0427 (9)	
O7	−0.2021 (4)	0.07356 (18)	0.2898 (5)	0.0309 (7)	
O8	0.4601 (7)	−0.0181 (3)	0.4257 (10)	0.0777 (18)	
H1W	0.553 (6)	−0.004 (6)	0.515 (11)	0.093*	
H2W	0.377 (7)	−0.006 (6)	0.451 (14)	0.093*	
H4W	0.105 (10)	0.080 (2)	0.551 (16)	0.093*	
H5W	0.092 (12)	−0.008 (5)	0.167 (10)	0.093*	
H6W	0.229 (5)	0.011 (5)	0.340 (12)	0.093*	
H7W	−0.214 (15)	0.067 (5)	0.393 (9)	0.093*	
H8W	−0.195 (16)	0.034 (3)	0.238 (13)	0.093*	
H3W	0.248 (3)	0.126 (4)	0.620 (19)	0.093*	

### Atomic displacement parameters (Å^2^)

**Table:**

	*U*^11^	*U*^22^	*U*^33^	*U*^12^	*U*^13^	*U*^23^
Cu1	0.0221 (3)	0.0153 (3)	0.0307 (3)	−0.0014 (2)	0.0111 (2)	0.0008 (2)
C1	0.024 (2)	0.021 (2)	0.033 (3)	0.0026 (14)	0.013 (2)	0.0011 (15)
C2	0.0229 (19)	0.0210 (17)	0.0315 (18)	0.0045 (18)	0.0084 (15)	−0.0004 (19)
C3	0.0223 (19)	0.0198 (18)	0.0229 (18)	0.0031 (15)	0.0083 (15)	0.0014 (14)
C4	0.0208 (18)	0.0218 (19)	0.0220 (17)	0.0000 (14)	0.0064 (15)	0.0016 (15)
C5	0.026 (2)	0.0141 (17)	0.032 (2)	0.0009 (13)	0.017 (2)	0.0013 (15)
C6	0.034 (3)	0.027 (2)	0.042 (3)	0.0042 (18)	0.022 (2)	0.0008 (16)
C7	0.045 (3)	0.0219 (18)	0.044 (2)	0.0067 (19)	0.025 (2)	0.0011 (18)
N1	0.0284 (18)	0.0215 (16)	0.0293 (17)	0.0018 (14)	0.0125 (15)	−0.0001 (14)
O1	0.0475 (19)	0.0275 (18)	0.0292 (16)	−0.0008 (15)	0.0133 (15)	0.0000 (14)
O2	0.055 (2)	0.0236 (14)	0.0343 (16)	−0.0049 (14)	0.0230 (16)	−0.0069 (13)
O3	0.0226 (17)	0.0253 (16)	0.0519 (19)	0.0004 (12)	0.0185 (14)	0.0011 (14)
O4	0.0274 (17)	0.0214 (15)	0.073 (2)	0.0041 (14)	0.0220 (17)	−0.0029 (19)
O5	0.0266 (16)	0.0281 (19)	0.033 (2)	−0.0011 (11)	0.0062 (16)	−0.0014 (12)
O6	0.0362 (17)	0.0247 (16)	0.056 (2)	0.0078 (15)	0.0077 (15)	−0.0088 (16)
O7	0.0313 (16)	0.0262 (15)	0.0355 (16)	−0.0074 (13)	0.0142 (14)	−0.0040 (14)
O8	0.035 (2)	0.054 (3)	0.108 (4)	0.0047 (18)	−0.006 (3)	−0.023 (3)

### Geometric parameters (Å, °)

**Table:**

Cu1—O7	2.061 (3)	C4—C1^ii^	1.525 (5)
Cu1—O6	2.068 (3)	C5—C6	1.325 (6)
Cu1—O3	2.071 (3)	C5—H5	0.9300
Cu1—O1	2.119 (4)	C6—C7	1.379 (7)
Cu1—O5	2.178 (5)	C6—H6	0.9300
Cu1—N1	2.187 (4)	C7—N1	1.350 (6)
C1—O1	1.228 (7)	C7—H7	0.9300
C1—O2	1.258 (6)	O5—H4W	0.82 (2)
C1—C4^i^	1.525 (5)	O5—H3W	0.82 (9)
C2—O4	1.227 (5)	O6—H5W	0.82 (7)
C2—O3	1.244 (6)	O6—H6W	0.83 (7)
C2—C3	1.552 (6)	O7—H7W	0.83 (8)
C3—N1	1.328 (5)	O7—H8W	0.83 (8)
C3—C4	1.399 (6)	O8—H1W	0.84 (8)
C4—C5	1.349 (6)	O8—H2W	0.83 (8)
			
O7—Cu1—O6	95.00 (16)	C3—C4—C1^ii^	123.2 (4)
O7—Cu1—O3	93.52 (13)	C6—C5—C4	117.5 (4)
O6—Cu1—O3	171.34 (14)	C6—C5—H5	121.2
O7—Cu1—O1	84.19 (14)	C4—C5—H5	121.2
O6—Cu1—O1	84.83 (16)	C5—C6—C7	121.3 (5)
O3—Cu1—O1	97.60 (15)	C5—C6—H6	119.3
O7—Cu1—O5	88.09 (15)	C7—C6—H6	119.3
O6—Cu1—O5	86.87 (16)	N1—C7—C6	121.4 (4)
O3—Cu1—O5	91.87 (14)	N1—C7—H7	119.3
O1—Cu1—O5	168.12 (14)	C6—C7—H7	119.3
O7—Cu1—N1	171.80 (13)	C3—N1—C7	118.0 (4)
O6—Cu1—N1	92.89 (17)	C3—N1—Cu1	110.5 (3)
O3—Cu1—N1	78.54 (13)	C7—N1—Cu1	131.3 (3)
O1—Cu1—N1	98.75 (15)	C1—O1—Cu1	145.4 (3)
O5—Cu1—N1	90.13 (15)	C2—O3—Cu1	117.2 (3)
O1—C1—O2	125.8 (4)	Cu1—O5—H4W	77 (8)
O1—C1—C4^i^	118.0 (4)	Cu1—O5—H3W	119 (10)
O2—C1—C4^i^	116.1 (4)	H4W—O5—H3W	114 (4)
O4—C2—O3	126.1 (5)	Cu1—O6—H5W	118 (7)
O4—C2—C3	117.0 (5)	Cu1—O6—H6W	122 (7)
O3—C2—C3	116.8 (4)	H5W—O6—H6W	114 (9)
N1—C3—C4	120.1 (4)	Cu1—O7—H7W	129 (8)
N1—C3—C2	115.9 (4)	Cu1—O7—H8W	92 (8)
C4—C3—C2	123.9 (4)	H7W—O7—H8W	112 (9)
C5—C4—C3	121.5 (4)	H1W—O8—H2W	111 (4)
C5—C4—C1^ii^	115.3 (4)		

Symmetry codes: (i) *x*−1/2, −*y*+1/2, *z*−1/2; (ii) *x*+1/2, −*y*+1/2, *z*+1/2.

### Hydrogen-bond geometry (Å, °)

**Table:**

*D*—H···*A*	*D*—H	H···*A*	*D*···*A*	*D*—H···*A*
O5—H3W···O4^ii^	0.82 (9)	1.93 (8)	2.735 (6)	167 (8)
O5—H4W···O6^iii^	0.82 (2)	2.35 (8)	2.966 (5)	132 (10)
O6—H5W···O5^iv^	0.82 (7)	2.29 (7)	2.966 (5)	140 (9)
O7—H7W···O2^v^	0.83 (8)	1.90 (9)	2.720 (5)	169 (8)
O7—H8W···O2^iii^	0.83 (8)	2.03 (4)	2.825 (5)	162 (11)

Symmetry codes: (ii) *x*+1/2, −*y*+1/2, *z*+1/2; (iii) *x*, −*y*, *z*+1/2; (iv) *x*, −*y*, *z*−1/2; (v) *x*, *y*, *z*+1.
